# Effect of food hygiene training on food handlers’ knowledge in Sokoto Metropolis: a quasi-experimental study

**DOI:** 10.11604/pamj.2021.40.146.27183

**Published:** 2021-11-09

**Authors:** Ismail Abdullateef Raji, Oche Mansur Oche, Aminu Umar Kaoje, Kehinde Joseph Awosan, Mansur Olayinka Raji, Godwin Jiya Gana, Jessica Timane Ango, Auwal Usman Abubakar

**Affiliations:** 1 Department of Community Medicine, Usmanu Danfodiyo University Teaching Hospital, Sokoto, Nigeria,; 2 Nigeria Field Epidemiology and Laboratory Training Program, 50 Haile Selassie Street, Asokoro, Abuja, Nigeria,; 3 Department of Community Health, Usmanu Danfodiyo University, Sokoto, Nigeria

**Keywords:** Training, intervention, food handlers, food hygiene, restaurants

## Abstract

**Introduction:**

training intervention for food handlers is necessary to increase their knowledge and awareness about food hygiene. Research in this area has been given low attention in Nigeria, especially in the Northern part of the country. Therefore, we assessed the effect of food hygiene training on the knowledge of food hygiene among food handlers in Sokoto metropolis.

**Methods:**

we conducted a quasi-experimental study between January and July 2019. We used a multistage sampling technique to select 360 food handlers randomized into intervention and control groups. We conducted a training intervention after the baseline data collection. Post-intervention data collection was conducted six months after the intervention. We estimated the proportion of respondents with good knowledge at baseline and post-intervention. We assessed the difference in pre-and post-intervention proportions using McNemars Marginal Homogeneity test at 5% level of significance.

**Results:**

in the intervention and control groups, 19 (10.6%) and 18 (10.0%) had primary education respectively, p = 0.231. At baseline, 23 (12.8%) and 22 (12.2%) in intervention and control groups respectively had good knowledge, p= 0.515. At post-intervention, the proportion of those with good knowledge in the intervention group increased to 56.7%, p < 0.001; while in the control group, there was no significant difference in the proportion of those with good knowledge, p = 0.248.

**Conclusion:**

the training intervention has significantly improved the knowledge of the food handlers. We recommend that the National Food and Drug Agency, in collaboration with restaurant owners, ensure regular on-the-job training of food handlers.

## Introduction

Foodborne Diseases (FBDs) remains a significant health problem in both developed and developing countries [1]. Globally, increases in the incidence of FBDs continue to be reported, often associated with FBD outbreaks that at times raise international concern [2]. It is estimated that about one-third of the population in the developed world suffers morbidity due to FBDs. In the developing world, close to 2 million mortality are recorded each year [2]. The millions of people who become sick each year and thousands who die are from consuming mishandled or contaminated foods [3].

A high proportion of FBD outbreaks are due to contamination by the food handlers spreading harmful organisms to a large group of people within a short time [4]. Therefore, their knowledge of hygiene in the food production chain plays a vital role in distributing harmful microorganisms and chemicals from the environment to the food items [5]. World Health Organization (WHO) estimates the global burden of FBDs to be 600 million every year from eating contaminated food, and 420 000 die every year [6, 7] resulting in loss of 33 million healthy life years (DALYs) [7]. Children under five years of age are particularly at high risk, with 125 000 children dying from FBDs every year. WHO African and South-East Asia regions have the highest burden of FBDs [7]. In these less developed regions, diarrhoeal diseases are the primary reason for mortality [5].

Studies conducted in developed and developing countries have found that most reported FBDs originated in food service establishments [8, 9]. Furthermore, studies on FBD risk factors have indicated that most outbreaks associated with food service establishments can be linked to food handlers´ improper food preparation practices [9, 10]. Urbanization and changes in consumer habits, including travel, have increased the number of people buying and eating food prepared in public places [6]. This is especially an increasing trend in developing countries like Nigeria [11]. Also, globalization has triggered growing consumer demand for a wider variety of foods outside our own culture, which are usually mostly obtained from fast food outlets or restaurants. As the demand for fast foods and eating in restaurants increases, this positively creates economic and business opportunities but also pose challenges for food safety [5].

Food handlers play an essential role in ensuring food safety throughout the chain of production, processing, storage and preparation [5]. For example, statistics show that as much as 60% of cases of food poisoning are caused by poor food handling technique and by contaminated food served in food service establishments [12]. Therefore, food safety depends mostly on food handlers and their level of knowledge and practice in the food production process, which plays a vital role in distributing harmful microorganisms from the environment to the food items [5].

Training intervention for food handlers is necessary to increase their knowledge and awareness about food hygiene, change their food handling and preparation behaviours, and ultimately, decrease the incidence and burden of FBD arising from poorly handled food. Studies have shown that the training of food handlers has a significant impact on their knowledge and practice of food hygiene [13-17]. However, research in this area has been given low attention in developing countries, including Nigeria. Although studies in Nigeria assessed knowledge of food hygiene among food handlers, [18-21] very few, if any, have assessed change in their knowledge following a training intervention. In light of these, there is a need to investigate the effect of food hygiene training on the knowledge of food hygiene among food handlers in Sokoto metropolis.

## Methods

**Study area:** Sokoto State has 23 Local Government Areas (LGAs), of which four are metropolitan. The restaurants within the metropolis get good patronage, as many civil servants and people involved in commercial activities get their meals from such outlets. Therefore, most of the restaurants in Sokoto State are located within the metropolis. Most of the raw materials used in restaurant food preparation are obtained from the main market (also called the meat and fish market). However, some also get their raw materials, especially those that buy in bulk, from the bush market (Kasuan Daji). Most restaurants are open between 9 a.m. and 8 p.m. Services are rendered to customers daily; however, only a few are operational on Sundays. The most typical method of preserving perishable foodstuffs in restaurants is the use of refrigerators or freezers. Most use modern equipment such as blenders and microwaves, while the commonest source of cooking energy is firewood.

**Study design:** a quasi-experimental design (with pre-and post-test design) was used.

**Inclusion and exclusion criteria:** we included commercial food handlers with at least six months of work experience. We excluded food handlers who intend to leave Sokoto within the training intervention period.

**Sample size estimation and sampling technique:** the minimum sample size of 180 per group was determined using the formula for comparing proportions [22]


$$n=\frac{2{\left(Z_{\alpha }+Z_{1-\beta }\right)}^{2}pq}{d^{2}}$$

with level of confidence (Z_α_) of 1.96, power (Z_1-β_) of 0.84, the proportion of food handlers with good knowledge in a previous study (p_1_) of 0.609 and a projected increase in knowledge post-intervention (p_2_) based on the hypothesis of 20% increase and mean proportion of knowledge p = 0.709, level of significance α = 0.05 and applying a design effect of 2.

We used a two-stage sampling technique to select the respondents. In stage one, two LGAs (Sokoto south and Wamakko) were selected from the four metropolitan LGAs in the state using simple random sampling by balloting. We used random allocation to allocate Wamakko to the intervention group and Sokoto South LGA to the control group. In stage two, we selected restaurants in the selected LGAs using simple random sampling, using a table of random numbers. Each restaurant in the selected LGA was considered as a cluster. All the food handlers who met the inclusion criteria were recruited into the study for every cluster selected. We continued this process until we got the required sample size for both intervention and control groups. A total of 20 and 23 restaurants were selected in the intervention and control LGAs, respectively.

**Study instruments:** the study instrument is an interviewer-administered questionnaire consisting of closed-ended questions. We adapted the questions that made up the questionnaire from different studies that assessed the knowledge of food hygiene [18, 20, 23-27]. The questions were suitably modified where necessary to fit the socio-cultural context of the study area. The questions were categorized into five key areas of food hygiene based on WHO five keys to safer food: hygiene, separating raw and cooked food, proper cooking, safe storage temperatures, and safe water and materials [23]. The questions were translated into the study area´s local language (Hausa). Android phones using Open Data Kit (ODK) software were used for data collection in the field by trained data collectors.

The research instrument was pre-tested on 36 purposively sampled food handlers (10% of sample size) in five randomly selected restaurants in one LGA (Dange Shuni) not selected for the study. We assessed the validity of the questionnaire by checking for content validity, and we checked the reliability by assessing the internal consistency of the questionnaire, Cronbach´s α = 0.710.

### Data collection

**Pre-intervention:** the pre-intervention data were collected from both groups using the study questionnaire. The pre-intervention data were collected just before the commencement of the food hygiene training for the intervention group. For the control group, no training was carried out after the collection of the first data. An identification card was given to any respondent that completed the questionnaire. This allowed for easy identification of respondents and the pairing of data at post-intervention. Phone numbers of each respondent were also collected for tracking purposes during the intervention period. For food handlers without phones, the phone numbers of restaurant owners were used instead.

**Intervention:** the intervention was food hygiene training. The WHO five keys to safer food manual for trainers [23] was adapted to suit the local context for the intervention. The training manual was translated into Hausa language. The chief researcher delivered the food hygiene training to the food handlers in the intervention group only. This was done at a selected location central to their work site to ensure full participation. Each training session involved a cluster of 30-40 respondents at a time. Therefore, there were six sessions within a week to cover for all respondents in the intervention group. Each training session lasted for 45 minutes, with 20-30 minutes of discussion and answering of questions. After a four-week interval, a second round of training was carried out to reinforce the information. The WHO Five Keys to Safer Food poster was printed into hand bills, which was distributed to respondents in the intervention group to reinforce the information. To ensure participation, transportation and meals were provided in each session for the respondents.

**Post-intervention:** the post-intervention survey was conducted in the intervention group six months after the initial data collection, and the control group's end-of-study data were also obtained six months after the initial data collection. The same instrument and research team were used for the post-intervention survey. Immediately after the post-intervention data collection, the control group was also given the same food hygiene training in the manner described above for the intervention group in order for them to also benefit from and acquire the training necessary for proper food handling in the interest of the public.

**Data analysis:** twenty-four questions were used to assess food hygiene knowledge. Each question had three possible responses: *yes, no*, and *don´t know*. Point values for each question were assigned as follows: correct response = 1, incorrect response = 0 and *don´t know* = 0. Scaled scores were computed by summing item responses. Scores on the total knowledge scale have a possible range of 0 to 24. Respondents´ knowledge was graded into good, fair and poor knowledge [28]. Those with scores ≥ 70% of the expected knowledge score were categorized into good knowledge, those with score 50-69% of expected knowledge score were categorized into fair knowledge and those < 50% of expected knowledge score were categorized into poor knowledge.

IBM® SPSS version 25 was used for analyses. Frequencies and proportions of sociodemographic variables and graded knowledge scores were computed. In addition, mean age and Standard Deviation (SD) was reported. Chi-square test and Fisher´s exact tests were used in determining if any significant differences exist in sociodemographic characteristics between the intervention and control groups. McNemar´s test was used to compare the proportion of respondents´ responses at baseline and post-intervention. McNemar´s marginal homogeneity test was used to assess differences in the proportion of respondents with overall good, fair or poor knowledge at baseline and post-intervention.

**Ethical consideration:** ethical approval for this study with reference number SKHREC/050/017 was obtained from the Sokoto State research ethics committee. Permission was received from the restaurant owners. The information sheet explaining the purpose and what the study entails was read and explained to the respondents to make an informed decision. The respondents were informed that they have the right to withdraw at any stage of the study if they so wish. They were informed that the research has no harm to their health; neither does it affect the security of their job if they choose not to participate in the research.

## Results

At the pre-intervention stage of the study, 360 questionnaires (180 per group) were administered to the respondents in the intervention and control groups with a 100% response rate. After the intervention, the study questionnaire was administered to 171 (95%) respondents who attended the two intervention sessions in the intervention group and 166 (92%) in the control group who were present for the End of Study (EOS) data collection.

The 25-34-year age group had the highest number of respondents in the intervention group, 64 (35.6%), while the 18-24-year age group had the highest number of respondents in the control group, 70 (35.6%). The age groups were similar, p = 0.071. The mean age in the intervention group was 31.8 ± 10.4 vs 28.8 ± 9.0 in the control group. This difference was not statistically significant, p = 0.05. In both groups, females were more than males; however, the control group had more females 159 (88.3%) than the control group, 118 (65.6%), the difference was statistically significant, p <0.0001. In the intervention and control groups, only 19 (10.6%) and 18 (10.0%) had primary education respectively, p = 0.231 (Table 1).

**Table 1:** socio-demographic profile of respondents

Variables	Intervention	Control	Test Statistics p-value
	n = 180	n = 180	
	n (%)	n (%)	
**Age group (years)**			
18-24	48(26.7)	70(38.9)	Fisher'Exact p = 0.071
25-34	64(35.6)	60(33.3)	
35-44	40(22.2)	35(19.4)	
45-54	23(12.8)	13(7.2)	
≥ 55	5(2.8)	2(1.1)	
**Gender**			
Female	118(65.6)	159(88.3)	χ^2^ = 26.322 **p < 0.001**
Male	62(34.4)	21(11.7)	
**Marital Status**			
Single	77(42.8)	107(59.4)	Fisher' Exact **p = 0.015**
Separated	1(0.6)	1(0.6)	
Divorced	10(5.6)	4(2.2)	
Widowed	17(9.4)	11(6.1)	
Married	75(41.7)	57(31.7)	
**Tribe**			
Hausa/Fulani	80(44.4)	61(33.9)	χ^2^ = 5.442 p = 0.142
Yoruba	34(18.9)	49(27.2)	
Ibo	14(7.8)	16(8.9)	
Others	52(28.9)	54(30.0)	
**Religion**			
Islam	136(75.6)	135(75.0)	χ^2^ = 0.015 p = 0.903
Christianity	44(24.4)	45(25.0)	
**Educational Level**			
None	25(13.9)	36(20.0)	χ^2^ = 5.596 p = 0.231
Quranic	47(26.1)	33(18.3)	
Primary	19(10.6)	18(10.0)	
Secondary	61(33.9)	57(31.7)	
Tertiary	28(15.6)	36(20.0)	
**Job description**			
Cooking	75 (41.7)	85(47.2)	χ^2^ = 1.125 p = 0.289
Serving/dish washing	105 (58.3)	95 (52.8)	
**Years of experience**			
<5	104 (57.8)	133(73.9)	χ^2^ = 10.386 **p = 0.001**
≥5	76 (42.2)	47(26.1)	
**Have you had food hygiene training**			
Yes	75(41.7)	65(36.1)	χ^2^ = 1.169 p = 0.280
No	105(58.3)	115(63.9)	

The job description was similar in both groups, p = 0.289. Most of the respondents had less than five years of work experience in the intervention and control groups. However, the control group had more respondents, 133 (73.9%) with years of experience less than five years than the intervention group, 104 (57.8%), p = 0.001. The proportion of respondents that have had food hygiene training were similar in both groups (75% and 65% respectively), p = 0.280 (Table 1).

**Figure 1:**
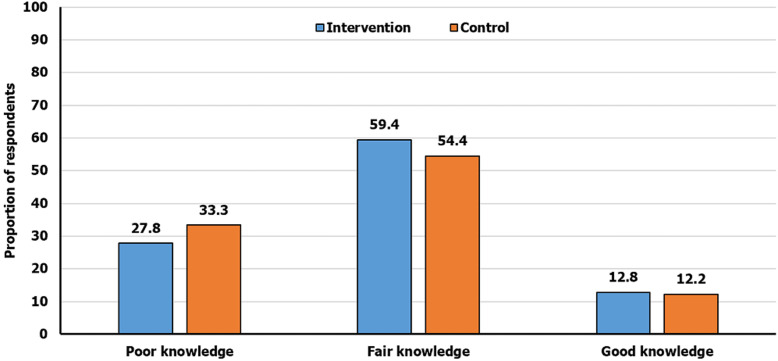
baseline overall knowledge of food hygiene

At baseline, only 23 (12.8%) and 22 (12.2%) in intervention and control groups respectively had good knowledge of food hygiene. The differences observed were not statistically significant, χ^2^ = 1.326, p = 0.515 (Figure 1).

At post-intervention, the proportion of those that knew it was important to wash hands before handling food increased from 95% to 97.7% in the intervention group. However, the change was not statistically significant, p = 0.125. The proportion of those that knew it was important to wash hands with soap increased from 91.1% to 93.0%; however, the increase was not statistically significant, p = 0.125. Finally, in the intervention group, the proportion of those who knew it was unhygienic to sneeze into hands increased from 41.7% to 55.6%, p < 0.001. In the control group, there was no significant difference in the proportion of respondents that had correct responses to variables that assessed knowledge of personal hygiene (Table 2).

**Table 2:** comparison of respondents’ knowledge of personal hygiene at pre-intervention and post-intervention

Variables	Intervention			Control		
	Pre-Int	Post-Int	Test stat* p-value	BOS	EOS	Test stat* p-value
	n = 180	n = 171		n = 180	n = 166	
	n (%)	n (%)		n (%)	n (%)	
Knew it is important to wash hands before handling food	171 (95.0)	167 (97.7)	χ^2^ = 0.800, p = 0.125	172 (95.6)	157 (94.6)	χ^2^ = 0.000 p = 1.000
Knew wiping cloths can spread microorganisms	142 (78.9)	154 (90.1)	χ^2^ = 14.450 **p < 0.001**	140 (77.8)	126 (75.9)	χ^2^ = 0.000 p = 1.000
Knew it necessary to use soap for hand washing	164 (91.1)	159 (93.0)	χ^2^ = 2.250 p = 0.125	161 (89.4)	142 (85.5)	χ^2^ = 3.200 p = 0.063
Knew the reason for drying hands after washing	23 (12.8)	69 (40.4)	χ^2^ = 45.021 **p < 0.001**	14 (7.8)	14 (8.4)	χ^2^ = 0.000 p = 1.000
Knew it is unhygienic to keep nails uncut	65 (36.1)	108 (63.2)	χ^2^ = 43.022 **p < 0.001**	77 (42.8)	68 (41.0)	χ^2^ = 0.000 p = 1.000
Knew it was unhygienic to sneeze into hands	75 (41.7)	95 (55.6)	χ^2^ = 19.360 **p < 0.001**	83 (46.1)	74 (44.6)	χ^2^ = 0.500 p = 0.500
Knew was unhygienic to cook without an apron	91 (50.6)	121 (70.8)	χ^2^ = 31.030 **p < 0.001**	105 (58.3)	96 (57.8)	χ^2^ = 0.500 p = 0.500
Knew it was unhygienic to wear jewellery when cooking	124 (68.9)	148 (86.5)	χ^2^ = 27.034 **p < 0.001**	122 (67.8)	111 (66.9)	χ^2^ = 0.000 p = 1.000
Knew proper cleaning of utensils decrease the risk of food contamination	106 (58.9)	118 (69.0)	χ^2^ = 12.500 **p < 0.001**	97 (53.9)	87 (52.4)	χ^2^ = 0.500 p = 0.500

* = McNemar's Chi square Test stat = Test Statistics **Pre-int** = pre-intervention **Post-int** = Post-intervention **BOS** = Beginning of study **EOS** = End of Study

In the intervention group, the proportion of respondents that knew it was wrong to use the same cutting board for raw and cooked food increased from 35.6% at pre-intervention compared to 56.1% at post-intervention, p < 0.001. In addition, the proportion of those that knew that raw food needs to be stored separately from cooked food (56.1% at pre-intervention and 80.1 at post-intervention); and raw meat cannot be stored above other foodstuffs in the refrigerator (45.6% at pre-intervention and 72.5% at post-intervention) increased significantly in the intervention group, p< 0.001. In the control group, there was no significant difference in results between the beginning and end of the study (Table 3).

**Table 3:** comparison of respondents’ knowledge of separating raw and cooked food and proper cooking of food, at pre-intervention and post-intervention

Variables	Intervention			Control		
	Pre-Int	Post- Int	Test stat* p-value	BOS	EOS	Test stat* p-value
	n = 180	n = 171		n = 180	n = 166	
	n (%)	n (%)		n (%)	n (%)	
Knew that the same cutting board should not be used for raw and cooked foods	64 (35.6)	96 (56.1)	χ^2^ = 36.026 **p < 0.001**	59 (32.8)	56 (33.7)	χ^2^ = 0.000 p = 1.000
Knew that raw food should be stored separately from cooked food	101 (56.1)	137 (80.1)	χ^2^ = 36.026 **p < 0.001**	110 (61.1)	99 (59.6)	χ^2^ = 0.000 p = 1.000
Knew that raw meat should not be stored above other foodstuffs in the refrigerator	82 (45.6)	124 (72.5)	χ^2^ = 42.188 **p < 0.001**	93 (51.7)	87 (52.4)	χ^2^ = 0.500 p = 0.500
Knew that it is unhygienic to cook in an unclean surrounding	118 (65.6)	142 (83.0)	χ^2^ = 29.032 **p <0.001**	121 (67.2)	55 (33.1)	χ^2^ = 0.000 p = 1.000
Knew that cooked foods need to be thoroughly reheated	76 (42.2)	92 (53.8)	χ^2^ = 29.032 **p <0.001**	56 (31.1)	53 (31.9)	χ^2^ = 0.000 p = 1.000
Knew that cooked food should be kept very hot before serving	128 (71.1)	149 (87.1)	χ^2^ = 25.037 **p <0.001**	134 (74.4)	120 (72.3)	χ^2^ = 1.333 p = 0.250
Knew that food is cooked on the outside may not necessarily be cooked on the inside	48 (26.7)	70 (40.9)	χ^2^ = 23.040 **p <0.001**	29 (16.1)	27 (16.3)	χ^2^ = 0.000 p = 1.000

* = McNemar's Chi square **Pre-int** = pre-intervention **Post-int** = Post-intervention **BOS** = Beginning of study **EOS** = End of Study

The proportion of those that knew refrigerating food only slowed bacterial growth increased from 48.9% to 76.6%, p < 0.001 at post-intervention. The proportion of respondents that knew that looks could not identify safe water increased from 18.9% to 36.3%, p < 0.001. The proportion of responses to questions addressing knowledge of safe cooking temperature, water use, and raw materials remained unchanged at the end of the study in the control group (Table 4).

**Table 4:** comparison of knowledge of safe cooking temperature, use of water and raw materials at pre-intervention and post-intervention

Variables	Intervention			Control		
	Pre-Int	Post- Int	Test stat* p-value	BOS	EOS	Test stat* p-value
	n = 180	n = 171		n = 180	n = 166	
	n (%)	n (%)		n (%)	n (%)	
Knew that refrigerating food only slows bacterial growth	88 (48.9)	131 (76.6)	χ^2^ = 44.033 **p < 0.001**	97 (53.9)	91 (54.8)	χ^2^ = 0.281 p = 0.597
Knew that improper storage of foods might be a hazard to health	129 (71.7)	130 (76.0)	χ^2^ = 3.125 p = 0.070	114 (63.3)	103 (62.0)	χ^2^ = 0.000 p = 1.000
Knew that it was not safe to cook when sick	124 (68.9)	127 (74.3)	χ^2^ = 4.000 **p = 0.039**	109 (60.6)	97 (58.4)	χ^2^ = 0.500 p = 0.500
Knew that wounds should be covered with waterproof dressing when cooking	32 (17.8)	64 (37.4)	χ^2^ = 35.027 **p < 0.001**	20 (11.1)	20 (12.0)	χ^2^ = 0.000 p = 1.000
Knew that only looks do not determine the safety of water	34 (18.9)	62 (36.3)	χ^2^ = 27.034 **p < 0.001**	30 (16.7)	28 (16.9)	χ^2^ = 0.500 p = 0.500
Knew that fruits and vegetables have to be always washed before consumption	151 (83.9)	148 (86.5)	χ^2^ = 3.200 p = 0.063	142 (78.9)	132 (79.5)	χ^2^ = 0.000 p = 1.000
Knew that food prepared in advance increases the risk of food contamination	59 (32.8)	76 (44.4)	χ^2^ = 13.474 **p < 0.001**	58 (32.2)	53 (31.9)	χ^2^ = 0.000 p = 1.000
Knew that using a cap, masks, protective gloves, and adequate clothing reduces the risk of food contamination	118 (65.6)	135 (78.9)	χ^2^ = 23.040 **p < 0.001**	120 (66.7)	111 (66.9)	χ^2^ = 0.000 p = 1.000

* = McNemar's Chi square **Pre-int** = pre-intervention **Post-int** = Post-intervention **BOS** = Beginning of study **EOS** = End of Study

**Figure 2:**
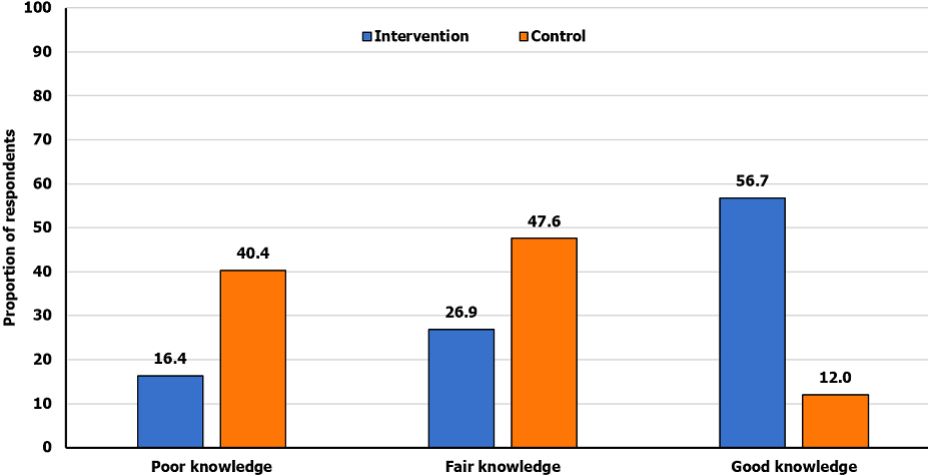
post-intervention overall knowledge of food hygiene

Following training intervention, the proportion of those with overall good knowledge in the intervention group increased from 12.0% to 56.7%. McNemars marginal homogeneity test showed that this was statistically significant, p <0.001. However, there was no significant difference in the proportion of those with good knowledge at EOS for the control group, p = 0.248 (Table 5). There was an appreciable difference in the proportion of those with poor knowledge in the intervention group (16.4%) vs the control group (40.4%). Similarly, there is a marked difference in the proportion of those with good knowledge in the intervention group (56.7%) compared to the control group (12.0%) after the intervention (Figure 2).

**Table 5:** comparison of overall knowledge at pre-intervention and post-intervention

Variables	Intervention	Control	Test Statistics p-value
	n = 180	n = 180	
	n (%)	n (%)	
**Age group (years)**			
18-24	48(26.7)	70(38.9)	Fisher'Exact p = 0.071
25-34	64(35.6)	60(33.3)	
35-44	40(22.2)	35(19.4)	
45-54	23(12.8)	13(7.2)	
≥ 55	5(2.8)	2(1.1)	
**Gender**			
Female	118(65.6)	159(88.3)	χ^2^ = 26.322 **p < 0.001**
Male	62(34.4)	21(11.7)	
**Marital Status**			
Single	77(42.8)	107(59.4)	Fisher' Exact **p = 0.015**
Separated	1(0.6)	1(0.6)	
Divorced	10(5.6)	4(2.2)	
Widowed	17(9.4)	11(6.1)	
Married	75(41.7)	57(31.7)	
**Tribe**			
Hausa/Fulani	80(44.4)	61(33.9)	χ^2^ = 5.442 p = 0.142
Yoruba	34(18.9)	49(27.2)	
Ibo	14(7.8)	16(8.9)	
Others	52(28.9)	54(30.0)	
**Religion**			
Islam	136(75.6)	135(75.0)	χ^2^ = 0.015 p = 0.903
Christianity	44(24.4)	45(25.0)	
**Educational Level**			
None	25(13.9)	36(20.0)	χ^2^ = 5.596 p = 0.231
Quranic	47(26.1)	33(18.3)	
Primary	19(10.6)	18(10.0)	
Secondary	61(33.9)	57(31.7)	
Tertiary	28(15.6)	36(20.0)	
**Job description**			
Cooking	75 (41.7)	85(47.2)	χ^2^ = 1.125 p = 0.289
Serving/dish washing	105 (58.3)	95 (52.8)	
**Years of experience**			
<5	104 (57.8)	133(73.9)	χ^2^ = 10.386 **p = 0.001**
≥5	76 (42.2)	47(26.1)	
**Have you had food hygiene training**			
Yes	75(41.7)	65(36.1)	χ^2^ = 1.169 p = 0.280
No	105(58.3)	115(63.9)	

## Discussion

This study was conducted to determine the effect of training intervention on food hygiene knowledge among food handlers in Sokoto metropolis. Respondents in both intervention and control groups were similar concerning most of the sociodemographic profiles except for gender and marital status in the control group with more females and more singles, respectively, and the differences were statistically significant.

Less than half of the respondents have had food hygiene training before the study. The likely explanation for this could be the lack of enforcement of food hygiene training by the State Primary Health Care Authority (SPHCDA). This finding could imply that food handlers are into the food business without fully understanding the implications of what their actions or inactions in food handling can be to consumers, especially within outbreaks of FBDs. Similar findings have been reported in previous studies in Nigeria, Ethiopia and Malaysia [19, 27, 29-31]. However, contrary to the finding in this study, studies in Ilorin and Abeokuta in Nigeria reported more than half of the food handlers had been trained on food hygiene [32, 33].

At baseline, there was poor knowledge of food hygiene in both intervention and control groups. This finding is not surprising because a significant proportion of respondents in both groups have never had any form of food hygiene training. The low proportion of respondents with overall good knowledge at baseline could be explained by the low knowledge of the respondents in various aspects that assessed knowledge. For example, less than half of the respondents in both study groups did not know that it is unhygienic to sneeze into hands. This is a public health concern as food handlers serve a significant portion of the community daily. Their poor knowledge and practices could lead to an unintentional FBD outbreak. The finding in this study is similar to what researchers have found in other parts of Nigeria, including Owerri in South East and Ijebu-Ode in South-West Nigeria [18, 20], Ethiopia [1] and Bangkok [27] where only a small proportion of the respondents had good knowledge of food hygiene. However, a contrary finding was obtained in studies in Nigeria, Ethiopia and Malaysia, where a high proportion of the respondents had good knowledge of food hygiene [19, 24, 34]. These studies were conducted in educational institutions, where more attention is more likely to be given to the knowledge of food handlers before they are employed in food service outlets. This could have been responsible for a higher proportion of people with good knowledge in these studies and, therefore, may not represent the general population of food handlers. However, with a high proportion of food handlers with good knowledge of food hygiene, lesser consumers are likely to be exposed to FBDs.

A high proportion of the respondents had correct responses to the variables that assessed personal hygiene in intervention and control groups. For example, most of the respondents knew the importance of handwashing before handling food; and washing hands with soap. This is a positive finding because knowledge of handwashing can reduce the risk of contaminating food. This finding is expected as exposure to media where advice and adverts on hand hygiene are common. Furthermore, most of the respondents have some form of formal education, and information such as handwashing before eating is taught in schools. This finding has a positive public health implication in preventing the transmission of FDBs. The findings are similar to what was obtained in a study in the Karimnagar district in India, where respondents knew that hands should be washed with soap and water before food preparation and serving [12].

Most cases of FBDs usually result from improper handling of food, including the inappropriate use of temperature during food preparation and conservation, cross-contamination, poor personal hygiene and inadequate food utensils. Therefore, poor knowledge about these areas may lead to an outbreak of FBDs [35]. Unfortunately, only a few variables that assessed the knowledge of proper cooking at baseline were answered correctly in both intervention and control groups. This indicates that they may not realize that food not prepared or reheated with the right temperature may contribute to the risk of FBDs. This finding has negative public health implications because poor knowledge of foods that require adequate cooking or heating could lead to the transmission of FBDs. Studies in Europe have reported similarly low proportions of respondents with correct responses to variables that assessed proper cooking temperature among food handlers [36, 37]. However, a contrary finding was obtained in a study in Malaysia, where a high proportion of the respondents knew the correct responses to questions that assessed proper temperature for cooking and reheating food [38].

Training and education are essential to ensure that food handlers have the awareness and knowledge necessary to comply with food hygiene demands. Following a training intervention in this study, the proportion of respondents who had good knowledge about food hygiene increased. The increase in the overall knowledge in the intervention group could be attributed mainly to the training intervention, considering that at baseline, the intervention and control groups were comparable. The findings in the intervention group are similar to findings from previous training intervention studies where there was a significant improvement in overall food hygiene knowledge following a training intervention [39-44].

We recognize that this study has some limitations. Guessing is a possibility when answering the questions. This may distort the accurate measure of food hygiene knowledge. To minimize guessing, a “don´t know” option was included in the list of responses for each question that assessed knowledge.

## Conclusion

In conclusion, this study has demonstrated that training intervention can significantly improve the knowledge of food handlers in food hygiene. Therefore, we recommend that the National Food and Drug Agency, in collaboration with restaurant owners, should ensure regular on-the-job training of food handlers best food hygiene practices.

### What is known about this topic

- A high proportion of FBD outbreaks are due to contamination by food handlers;
- Knowledge of food hygiene has been well studied in Nigeria

### What this study adds

- At baseline, there was poor knowledge of food hygiene in both intervention and control groups;
- Following the training interventions, there was a significant increase in the proportion of those with good knowledge of food hygiene.
